# Autologous platelet-rich plasma versus autologous serum in treatment of dry eye disease

**DOI:** 10.1007/s00417-026-07170-y

**Published:** 2026-03-31

**Authors:** Sameh S. Mandour, Hatem Mariey, Ahmed Elgyoshy, Ahmed Shebl Fayed

**Affiliations:** Ophthalmology Department, Faculty of Medicine, Menoufia University, Shebin El-Kom, Menoufia, Egypt

**Keywords:** Dry eye disease, Platelet-rich plasma, Autologous serum

## Abstract

**Purpose:**

This work aimed to assess the efficacy and safety of autologous PRP versus AS in treatment of moderate to severe DED.

**Methods:**

A prospective, comparative, randomized controlled clinical trial involved 96 eyes of 48 cases with moderate and severe DED divided into 3 groups (16 patients each). Group 1 received PRP eye drops, Group 2 received AS eye drops and Group 3 (control group) received sodium hyaluronate eye drops. All participants were subjected to Ocular Surface Disease Index (OSDI), tear film break-up time (TBUT), Schirmer 1 test, ocular surface staining, and best corrected visual acuity (BCVA).

**Results:**

PRP and AS showed significant improvements in all parameters compared to sodium hyaluronate, with greater improvement in OSDI, Schirmer test and ocular surface staining with PRP compared to AS.

**Conclusion:**

PRP and AS have high safety and efficacy rates in moderate and severe DED, providing better symptom relief and clinical improvements than artificial tears with superiority for PRP over AS.

## Abstract (highlights)

**Key messages:**

***What is known*:**

- ◦ Artificial tears are sometimes ineffective in cases of moderate & severe dry eye.
- ◦ Autologous platelet rich plasma & autologous serum are promising more effective treatments in these cases.

***What is new*:**

- ◦ Autologous platelet rich plasma may have a superior effect to autologous serum.
- ◦ Autologous serum is effective and safe at 100% concentration.

## Introduction

Dry eye disease (DED) represents a highly prevalent eye disorder, which accounts for 25% of patients visiting ophthalmic clinics [1]. The etiologies of DED are diverse, with multiple risk factors including advancing age, postmenopausal stages in women, laser refractive surgeries, and prolonged contact lens use [2]. Recently, DED is defined as a complex disorder including ocular surface inflammation with instability and raised osmolarity of the tears leading to eye discomfort, vision impairment and possible ocular surface damage [3].

Standard treatment of DED primarily focuses on compensating defective tears by artificial substitutes. However, unsatisfactory or ineffective results are commonly obtained, particularly in severe cases. This has led to exploration of alternative therapies that aim to restore ocular surface such as those based on blood derivatives through their regenerative capacity in tissue repair [4]. Unlike artificial tears, autologous serum (AS) resembles natural tears in pH, osmolarity, and vital components like trophic factors, vitamins and bacteriostatic products as lysozyme and complement making it more beneficial in DED [5]. Furthermore, plasma-derived products like platelet rich plasma (PRP) also showed promising results due to their higher platelet concentrations that contain massive stores of growth factors, thus more powerfully promote cell proliferation and wound healing [4, 6]. This study aimed to assess the efficacy and safety of autologous Platelet Rich Plasma (PRP) versus autologous serum (AS) in management of moderate to severe DED.

## Patients and methods

A prospective comparative single blind randomized controlled clinical trial was conducted on 96 eyes of 48 patients with moderate and severe DED who attended ophthalmology outpatient clinic of Menoufia University hospital between November 2023 & October 2024. The sample size for the study was calculated using Statistics and Sample size pro program version 6 and the least sample size was found to be 32 eyes for each studied group using an alpha level of 0.05 and a study power of 80%.

All patients agreed to participate in the study and signed informed written consents. Approval of Menoufia Faculty of Medicine’s local Ethical Research Committee was obtained and the study was carried out in accordance with the principles of the declaration of Helsenki. The inclusion criteria included moderate DED with OSDI score 23–32 points and severe DED with OSDI score 33–100 points, while exclusion criteria included normal ocular surface (OSDI score 0–12 points), mild DED (OSDI score 13–22 points) and other abnormal ocular surface conditions like corneal abrasions, pterygium and ectropion.

All patients underwent a comprehensive assessment, including thorough history taking, assessment of DED severity by ocular surface disease index (OSDI) questionnaire [7, 8], recording best corrected visual acuity (BCVA) using decimal notation and slit lamp examination. Fluorescein tear break-up time (TBUT) [9] was performed by inserting fluorescein strip into the lower fornix followed by tear film examination using cobalt blue filter and recording the time until the first break up area appeared. Schirmer 1 test (without anaesthesia) [10] was carried out by measuring the wetting of scaled filter paper inserted into the lower fornix. Ocular surface staining with fluorescein stain was graded using modified Oxford scale [11].

The patients were randomly divided into 3 groups; each included 32 eyes of 16 patients: **Group 1** received autologous PRP eye drops 4 times daily for 6 weeks. **Group 2** received AS eye drops 4 times daily for 6 weeks. **Group 3 (Control)** received preservative-free sodium hyaluronate artificial tears eye drops 4 times daily for 6 weeks. Eligible patients were randomly allocated (1:1:1) to either the PRP, AS or Control group using a computer-generated block randomization sequence.

### **PRP eye drops preparation** [12]

9 ml of blood was drawn from the patient to a sterile tube containing sodium citrate as anticoagulant and was centrifuged with single-spin method at 2500 revolutions/min (RPM) for 3 min then the supernatant PRP was extracted and provided immediately for use by the patients. Patients were instructed to use the vial for one week, storing it refrigerated at + 4–8 °C between uses. The preparations were repeated weekly over the total treatment period of 6 weeks.

### AS eye drops preparation [5]

10 ml of blood was drawn from the patient to an empty sterile tube and kept at room temperature for two hours until complete clotting occurred then the blood was centrifuged at 3000 RPM for 15 min to segregate the serum from solid elements then the serum was extracted and provided immediately for use by the patients. Patients were instructed to use the vial for one week, storing it refrigerated at + 4–8 °C between uses. The preparations were repeated weekly over the total treatment period of 6 weeks.

The key difference in the preparation of AS compared to PRP was the timing of the coagulation process and the presence of leukocytes [13]. During AS preparation, coagulation occurred spontaneously, and in presence of leukocytes, whereas in PRP, coagulation did not occur during the production but it was induced at site of application resulting in double concentration of platelets which led to slower release and higher concentration of growth factors in PRP, without the pro-inflammatory cytokines associated with leukocytes in AS [14].

After 6 weeks of treatment, OSDI score, TBUT, Schirmer 1 test, ocular surface staining by modified Oxford scale, and BCVA were re-assessed to evaluate treatment outcomes. Patients were assessed during treatment period to detect any side effects and ensure treatment safety.

### Statistical Analysis

The data were analyzed using Statistical Package for the Social Sciences SPSS program version 26.0 (IBM Corporation, Armonk, NY, USA). Descriptive statistics included numbers & percentages for qualitative variables and means ± standard deviations (SD) for quantitative variables. As for analytic statistics, chi-squared test was used to find association between two or more qualitative variables. Student’s t-test was used to compare two normally distributed quantitative variables, while Mann-Whitney test was used to compare two non-normally distributed quantitative variables. ANOVA test was used to compare more than two normally distributed quantitative variables. Paired t test compared two normally distributed related samples while Wilcoxon test compared two non-normally distributed related samples. Shapiro-Wilks test was used to assess normality. Statistical significance was achieved when P-value ≤ 0.05.

## Results

This study included 48 patients. Their ages ranged from 45 to 78 years, with a mean of 58.27 years ± 7.73 years standard deviation (SD). They included 30 females (62.5%) and 18 males (37.5%). Table (1) shows the demographic data of the study groups.

**Table 1:** Demographic data among the study groups

Demographic data	PRP (*n* = 16 patients)	AS (*n* = 16 patients)	Control (*n* = 16 patients)	Test	*P*- value
**Age: (years)** ▪ Mean ± SD ▪ Range ▪ Median	58.37 ± 7.81 45–71 59.5	58.37 ± 9.17 47–78 56.5	58.06 ± 6.48 50–70 57	F test 1.04	0.992
**Gender: n (%)** ▪ Male ▪ Female	7 (43.8) 9 (56.3)	5 (31.3) 11 (68.8)	6 (37.5) 10 (62.5)	χ^2^ 0.533	0.766

SD: Standard deviation -χ^2^: Chi-squared Test -F: One-way ANOVA test

Severity of DED was determined by OSDI score. Fifty six eyes (58.3%) had moderate DED (OSDI score 23–32) and 40 eyes (41.7%) had severe DED (OSDI score ≥ 33).

Pre-treatment, there were no statistically significant differences in baseline values of all parameters between the study groups. After 6 weeks of treatment, OSDI score decreased significantly in all groups (*P* < 0.001), with all members of the 3 groups achieving more than 6-pont improvement. Improvement was more significant in PRP and AS groups compared to the control group (P2 < 0.001, P3 < 0.001 respectively), and more significant improvement was observed in in PRP group compared to AS group (P1 < 0.001) (Table 2).

**Table 2:** OSDI pre and 6 weeks post treatment in the study groups

Variables	PRP (*n* = 16 patients)	AS (*n* = 16 patients)	Control (*n* = 16 patients)	Test	*P*-value
**OSDI pre** ▪ Mean ± SD ▪ Range ▪ Median	68.1 ± 14.59 50–87.5 68.7	68.17 ± 10.43 50–84.3 68.95	71.47 ± 14.18 52.7–87.5 69.4	t-test 0.01 0.86 0.73	P1 = 0.987 P2 = 0.541 P3 = 0.471
**OSDI post** ▪ Mean ± SD ▪ Range ▪ Median	30 ± 3.13 25–35 30	36.37 ± 3.13 30–39 37	51.31 ± 8.47 38–64.5 52.7	t-test 0.61 8.82 6.56	P1 < 0.001** P2 < 0.001** P3 < 0.001**
**P-value** (pre & post)	< 0.001**	< 0.001**	< 0.001**		

t=independent student t test **=highly significant, P1 = p value between PRP and AS groups, P2 = p value between PRP and control groups, P3 = p value between AS and control groups

Table 3 shows TBUT results. Significant improvement was observed in both of PRP and AS groups post-treatment, with no significant difference between the two groups (P1 = 0.19). No significant improvement was observed in the control group.

**Table 3:** TBUT pre and 6 weeks post treatment in the study groups

Variables	PRP (*n* = 32 eyes)	AS (*n* = 32 eyes)	Control (*n* = 32 eyes)	Test	*P*- value
**TBUT pre** ▪ Mean ± SD ▪ Range ▪ Median	4.44 ± 1.93 2–7 3.5	5.12 ± 1.96 2–9 5	4.46 ± 1.48 2–7 4.5	U 1.38 0.18 1.28	P1 = 0.17 P2 = 0.86 P3 = 0.20
**TBUT post** ▪ Mean ± SD ▪ Range ▪ Median	7.75 ± 1.68 5–10 8	7.18 ± 1.35 6–10 7	4.56 ± 1.38 2–7 4.5	U 1.3 5.8 5.7	P1 = 0.19 P2 < 0.001** P3 < 0.001**
**P-value** (pre & post)	< 0.001**	< 0.001**	0.083		

U=Mann-Whitney U test **=highly significant, P1 = p value between PRP and AS groups, P2 = p value between PRP and control groups, P3 = p value between AS and control groups

Schirmer 1 test results are shown in Table 4. Significant improvement was observed in both PRP and AS groups post-treatment, with significantly greater improvement in the PRP group (P1 = 0.04). However, no significant improvement was recorded in the control group.

**Table 4:** Schirmer 1 test pre and 6 weeks post treatment in the study groups

Variables	PRP (*n* = 32 eyes)	AS (*n* = 32 eyes)	Control (*n* = 32 eyes)	Test	*P*- value
**Schirmer 1 pre** ▪ Mean ± SD ▪ Range ▪ Median	5.53 ± 2.09 2–9 5	5.69 ± 1.99 2–9 5.5	5.59 ± 1.97 1–8 6	U 0.37 0.21 0.05	P1 = 0.71 P2 = 0.83 P3 = 0.96
**Schirmer 1 post** ▪ Mean ± SD ▪ Range ▪ Median	8.65 ± 2.22 5–12 9	7.50 ± 2.03 3–11 8	5.68 ± 1.87 1–8 6	U 2.05 4.67 3.29	P1 = 0.04* P2 < 0.001** P3 = 0.001*
**P-value** (pre & post)	< 0.001**	< 0.001**	0.083		

U=Mann-Whitney U test **=highly significant *=significant, P1 = p value between PRP and AS groups, P2 = p value between PRP and control groups, P3 = p value between AS and control groups

Table 5 shows ocular surface staining results. Oxford score decreased significantly post-treatment in all groups. PRP group showed more significant improvement than AS group (P1 < 0.001) and both groups showed more significant improvement than control group (P2 < 0.001, P3 = 0.001 respectively).

**Table 5:** Ocular surface staining pre and 6 weeks post-treatment in the study groups

Variables	PRP (*n* = 32 eyes)	AS (*n* = 32 eyes)	Control (*n* = 32 eyes)	Test	*P*- value
**Oxford score (pre)** ▪ Mean ± SD ▪ Range ▪ Median	2.71 ± 0.91 1–5 3	2.75 ± 0.88 2–5 3	3.0 ± 1.05 2–5 3	U 0.06 0.83 0.89	P1 = 0.95 P2 = 0.41 P3 = 0.37
**Oxford score (post)** ▪ Mean ± SD ▪ Range ▪ Median	0.71 ± 0.68 0–2 1	1.84 ± 0.99 1–5 2	2.71 ± 1.08 1–5 2	U 4.67 6.34 3.42	P1 < 0.001** P2 < 0.001** P3 = 0.001*
**P-value** (pre & post)	< 0.001**	< 0.001**	0.003*		

U=Mann-Whitney U test **=highly significant, *=significant, P1 = p value between PRP and AS groups, P2 = p value between PRP and control groups, P3 = p value between AS and control groups

Table 6 shows BCVA in the study groups. All groups showed significant improvement post-treatment, which was more significant in PRP and AS groups compared to control group (P2 < 0.001, P3 = 0.02 respectively). There was no significant difference between PRP & AS groups (P1 = 0.10).

**Table 6:** BCVA pre and 6 weeks post treatment in the study groups

Variables	PRP (*n* = 32 eyes)	AS (*n* = 32 eyes)	Control (*n* = 32 eyes)	Test	*P*- value
**BCVA pre** ▪ Mean ± SD ▪ Range ▪ Median	0.34 ± 0.15 0.1–0.6 0.3	0.34 ± 0.15 0.16–0.6 0.3	0.32 ± 0.14 0.1–0.5 0.3	U 0.10 0.57 0.52	P1 = 0.92 P2 = 0.57 P3 = 0.60
**BCVA post** ▪ Mean ± SD ▪ Range ▪ Median	0.53 ± 0.17 0.16- 1 0.5	0.46 ± 0.14 0.24–0.6 0.5	0.38 ± 0.15 0.1–0.6 0.42	U 1.66 3.48 2.19	P1 = 0.10 P2 < 0.001** P3 = 0.02*
**P-value** (pre & post)	< 0.001**	< 0.001**	0.001*		

U=Mann-Whitney U test **=highly significant, *=significant, P1 = p value between PRP and AS groups, P2 = p value between PRP and control groups, P3 = p value between AS and control groups

Regarding treatment safety, PRP and control groups didn’t record any side effects during treatment. Two eyes of one patient (6.25%) had mild discomfort with AS treatment but compared to others groups, the difference was statistically insignificant (*P* = 0.146) (Table 7).

**Table 7:** Side effects during treatment in the study groups

Side effects	PRP *N* (%)	AS *N* (%)	Control *N* (%)	Test χ2	*P*-Value
**Ocular discomfort**	0 (0)	2 (6.25%)	0 (0)	3.84	0.146

χ^2^: Chi-squared test

## Discussion

In this study PRP was extracted by centrifuging blood weekly by single spin for 3 min at 2500 RPM and kept at + 4–8 °C. This technique was described in a previous study, reporting that it produced a significant increase in platelets count with 1.90 enrichment [12]. While Alio et al. [14] used 1400 RPM for 10 min giving 1.71 enrichment and significant decrease of RBCs and leukocytes by 99.5% and 81.2%, respectively.

For AS preparation there are no standard protocols to achieve the desired therapeutic effects [15]. The leukocytes and pro-inflammatory cytokines present during preparation, including transforming growth factor (TGF-β) which delays corneal healing [16], favor diluting AS to 20% to match its physiologic levels in natural tears. However this results in decreasing growth factors concentration and reducing its efficacy [17]. Nevertheless, many trials recorded great results without side effects with 50% and 100% AS [18]. Liu et al. [5] reported that 2 h clotting and 3000 RPM centrifugation rate for 15 min increased concentrations of many trophic substances like epidermal growth factor (EGF), while significantly decreased TGF-β levels in AS. In our study AS was prepared similarly but without dilution as 100% serum to obtain advantages of fully concentrated therapy.

Studies that investigated the concentration of trophic factors within PRP and AS showed significantly higher concentrations in PRP [4] with levels of some growth factors being increased with storage. Metheetrairut et al. [19] reported that fibronectin and EGF were found with greater levels in 20% PRP and significantly increased after one week storage at 4 °C, while they were stable after storage in 20% AS. Wróbel-Dudzińska et al. [20] found significant increase in platelets count from 194.23 × 10^3^/µL in whole blood to 297.058 × 10^3^/µL in PRP with 1.5 enrichment. Levels of Fibroblast growth factor (FGF), fibronectin, EGF and substance p were significantly higher in PRP; however, TGF-β was more concentrated in AS.

In our study PRP significantly improved OSDI scores, TBUT, Schirmer test values & ocular surface staining scores. Similar results were recorded by Alio et al. [6] where 89% of cases improved in symptoms and 56% showed better tear film quality after 4 weeks. Another study showed symptomatic improvement in 87.5% of cases as well as improved Schirmer values after 6 weeks [21]. Also, Garcia et al. [22] reported greater improvements in all parameters with PRP compared to sodium hyaluronate. Marina et al. [23] reported better OSDI scores and Schirmer outcomes than ours, most probably due to using double-spin method in preparing PRP, which produced more enriched but lower volume product. Conversely, our results were better than Alio et al. [6] studies in corneal staining and BCVA, probably due to more enriched and fresh PRP with longer treatment duration.

Regarding AS, our study reported significant improvement in OSDI scores, TBUT, Schirmer and ocular surface staining scores. Comparable results were recorded by Kojima et al. [24] who reported improvements in Sjogren Syndrome (SS) and non-SS patients with AS. Noble et al. [25] and Urzua et al. [26] obtained significant improvement in OSDI scores using 50% and 20% AS with insignificant TBUT and corneal staining results, which may be attributed to using diluted AS preparation. Cho et al. [27] reported better improvements of symptoms and corneal staining in SS with 100% than 50% AS.

Comparing results of PRP and AS in our study, more significant decrease of OSDI score was observed in PRP group. This was aligned with study of Wróbel-Dudzińska et al. [20] where PRP reduced OSDI scores in all patients compared to 77.3% of patients in AS group. On the other hand, Metheetrairut et al. [19] and Jongkhajornpong et al. [28] found insignificant difference in OSDI scores between the 2 treatments.

In our study, both PRP & AS achieved significant improvements in TBUT with no significant difference between the 2 treatments. This matched the results of Kang et al. [29] and Özlük et al. [30]. Wróbel-Dudzińska et al. [20] and Jongkhajornpong et al. [28] also reported insignificant difference between PRP & AS regarding TBUT.

In our study, Schirmer’s test improvement was significantly higher in PRP group. PRP has been reported to have a stronger regenerative effect on acinar cells of lacrimal glands [31]. Metheetrairut et al. [19] reported that the changes observed in Schirmer’s test outcome were significantly better with PRP than with AS. On the other hand, Wróbel-Dudzińska et al. [20] and Jongkhajornpong et al. [28] observed insignificant difference in Schirmer test results between the 2 treatment groups. Passilongo et al. [32] and Özlük et al. [30] didn’t identify notable differences between the 2 groups.

In our study, there was a significantly greater improvement in ocular surface staining expressed as Oxford grading score in PRP group than AS group. Similar result was reported by Passilongo et al. [32]. However, Wróbel-Dudzińska et al. [20] & Jongkhajornpong et al. [28] noted no significant difference between the 2 treatments. Kang et al. [29] and Özlük et al. [30] also reported insignificant difference between both therapies.

Regarding their impact on visual acuity, our study showed a considerable improvement in BCVA after both PRP & AS treatment, with no significant difference between the 2 groups. Wróbel-Dudzińska et al. [20] reported improved BCVA with PRP in 45.5% of patients versus 13.6% with AS. Metheetrairut et al. [19] and Özlük et al. [30] reported that only PRP produced significant visual acuity improvement.

As for treatment safety in our study, no adverse effects were reported by PRP treated patients while one patient treated with AS reported mild discomfort which resolved spontaneously. This may be attributed to leucocytes and pro-inflammatory cytokines in 100% AS. Dalmon et al. [33] found that only 2 of 30 patients felt transient irritation with 100% AS while Noble et al. [25] found no side effects with 50% AS.

The current study emphasizes that both PRP and AS have shown promising results in treatment of moderate to severe DED, producing greater symptomatic improvement and better tear film quality than most commercially available artificial tears, while maintaining a high safety profile. Our study along with other studies, have demonstrated a relatively superior regenerative effect of PRP over AS, while other studies have shown comparable outcomes for the 2 preparations. Possible reasons for these discrepancies include different preparation protocols that influence cellular and growth factor content and stability, using different concentrations of AS that may affect its efficacy, as well as the demographic variations between the different study populations and the variable etiologies of DED in the enrolled study subjects.

This study has some limitations. The study was designed as a single-blind trial. While the members of the control group received preservative free sodium hyaluronate artificial tears eye drops with open label, the patients in the other 2 study groups were blinded to the type of blood derived product they received (PRP or AS). However, the evaluator performing the clinical measurements was not blinded. Members of the control group were not blinded to avoid unnecessary invasive procedures, since proper blinding of the control group would have necessitated the repeated withdrawal of blood samples from each subject. We recognize that this is a limitation, since the awareness of the treatment by the patient and/or evaluator might have introduced a significant bias, particularly in subjective endpoints (e.g. OSDI).

Additionally, we did not study the etiologies of DED in the study participants. The lack of matching of the study groups regarding DED etiology might have influenced the response to the treatments. Another limitation is that the single-spin protocols for PRP preparation generally produce lower platelet enrichment factors and less controlled red blood cells and leucocytes content compared to double-spin approaches. However, they offer some practical advantages including simplified technique, reduced processing time, and lower risk of contamination due to minimal handling [34].

## Conclusion

Both PRP and AS are safe, preservative-free, cost-effective treatments for moderate and severe DED. PRP may have a superior regenerative effect, resulting in greater improvement in OSDI score, Schirmer 1 test and ocular surface staining. AS at 100% concentration achieves high efficacy & is safe with no significant side effects.
